# A Little of Several Things

**Published:** 1902-06-15

**Authors:** Charles H. Warboys

**Affiliations:** Albion, Mich.


### A LITTLE OF SEVERAL THINGS.

BY DR. CHARLES H. WARBOYS, D.D.S., ALBION, MICH.

Read before the Southwestern Michigan Dental Society, April 9, 1902.

*Mr. President and Gentlemen:*—In this paper it will be my endeavor to mention a few things that I have found to be helpful to me, which I have adopted from the practice of other men.

The first thing, in operative procedures is the application of the rubber dam. On the incisors, cuspids and bi-cuspids, the ligature need seldom be used to hold the dam in place or to prevent leaking, by using a sufficiently large piece and placing the holes about as far apart as the teeth are from center to center, and turning the edge up under the gum with the ligature or a smooth instrument, it will hold and exclude moisture perfectly.

---

In the preparation of cavities, one should never hesitate to cut away all weak walls of enamel, after which the cavity may be formed so as to hold the filling, and no cavity will ever be found that can not be given a retaining form; of course it would be much better to crown some of them. I believe that the time will come when every cavity after being excavated, will be disinfected so thoroughly that there will not be a single germ left in it, or the tubuli of the tooth. A filling placed in such a cavity and one that fits so tightly as to be moisture-proof and remains so, will prevent the recurrence of decay. Until this is accomplished, we may expect recurrence of decay, no matter how well the filling is made.

I am a thorough believer in extension for prevention and restoration, and practice it. I look for the science of Bacteriaology to solve the problem of disinfection of the teeth that have been partially destroyed by the acids formed by the bacilli that are found in the mouth. Then we will not be so discouraged with our work of restoring the natural organs to their original form.

---

When introducing a gold filling into a cavity, where it has to withstand the stress of mastication and occlusion, I find that the gold must be built solidly into the retaining form, and in all cases should be constructed of cohesive gold to secure the best results.

The instruments that are being passed around are a few that have been remodeled from some that have been doing business fifty years or more (not by me, however). These are fashioned after those of Dr. Henry F. Libby, of Boston, whose three papers on his method were published in Vol. 40, of the *Cosmos*, and are very explicit. I am just trying the method, and am very well satisfied that it is worth becoming familiar with.

Those instruments are used to press or burnish soft or cohesive gold into the cavity and especially against the border or into undercuts. They do the work in many cavities, much better than any plugger that I ever used, and there is no danger of marring the borders and you are sure that the gold is going where it belongs. I am satisfied that with those instruments, I can force the gold against the mesio-occlusal border of a cavity in a bicuspid or molar much better than I can with a plugger that is malleted. Lately I have ground some of my pluggers perfectly smooth, and like them better than when they were serated. Any gold foil that is pure and carefully annealed is cohesive, and will work as well under a smooth instrument as a rough one.

I again refer you to Dr. Libby's papers, because you will there get the results of twenty-five years work.

---

In the construction of seamless crowns, the use of cement is preferable to plaster for the model, as it is stronger and can be polished after it is carved. A fine vent hole put through the model assures a perfect cast of it, when it is dipped into the hot metal.

---

Gold or copper plate heated to redness and then dropped into alcohol, will be softer and cleaner than when water is used.

---

For strengthening lower partial rubber plates, I have adopted the use of aluminum plate. After opening the flask and removing the wax, a piece of the lead is pressed around to fit the plaster that forms the lingual surface of the plate, and should extend one-fourth of an inch beyond the first tooth on the plate. This is used as a pattern to cut the aluminum by. After cutting the aluminum, it should be formed to fit the plaster by bending it with the thumbs and fingers. On the convex side of the metal a few loops may be soldered and then pressed to place, the case packed with rubber as usual, the aluminum appearing on the lingual side of the plate when it is completed.

Here is a formula for aluminum solder and flux.

| | | |
|---|---|---|
| ℞ | Aluminum plate - - | 6 parts. |
| | Zinc - - - - | 1 part. |
| | Phosphor tin - - - | 3 parts. |
| | Flux. Steric acid. | |

This solder is very useful for strengthening seamless aluminum crowns to prevent their wearing through on the occlusal surfaces.

In the construction of artificial dentures, judged from an artistic stand-point, I think we are very nearly rank failures. How often do we see a set of artificial teeth that look like natural ones? Are we influenced by the patients and their neighbors, or don't we know how to make them as they should be made? It is my opinion, that we are lacking in artistic conceptions more than in mechanical skill.

There are three mistakes that are very generally made, and to correct which, we are very poorly equipped when the mouth is edentulous. The color, form and size of the teeth for such a case is a matter of guess work for the most part. If the lower incisors remain we are all right, for then we have the color and form, the size can be had with the dividers as the two centrals and the lateral give us the exact width of the two upper centrals and the rest of the uppers are usually about right. This rule can be relied upon.

I wish that we could have a book with illustrations of a thousand or more different faces, life size and ages from thirty to sixty years, giving the color of the skin, eyes, hair and teeth, the latter to be matched with a standard shade ring, and illustrations from photographs of models of the teeth, these with a general description, and the various temperaments grouped and classified, would certainly aid us in no small way, in restoring the features of our patients.

The material for such a book could be gotten together in any of our large dental colleges, and the value of such a work would be almost unlimited to those of us who construct artificial dentures.

DISCUSSION.

Dr. N. S. Hoff: The essayist in the last suggestion of his paper makes a proposition which if it were carried out would no doubt be of great help to the prosthetist. But it would not be so easy a task as he seems to think, to gather and classify and then reproduce in colored print the various items which go to make up the human face artistically, when there is so little data upon which to start. To my mind it is not much to be surprised at that there are so few really artistic prosthetic dentures made to-day. It may be that we do not know how, as the essayist suggests; but, when we stop to consider the measly remuneration one gets for such services, how are we to expect that any one with artistic talent or a sufficiently high order of skill, such as is required to produce ideal work of this character, is going to sacrifice his opportunities for gaining a decent livelyhood in other branches, where his talent and skill are more appreciated. Unfortunately the mass of people who require such work do not encourage a high order of ability. But is it not probable that the dentists are perhaps more at fault in this matter than the patients? There is hardly one man out of five that really cares to do ordinary prosthetic work, and we find that those who can fill up their time in other lines of work, are very apt to let prosthetic work go by. There is no more interesting line of work in dentistry, and none that demands a higher grade of skill to successfully accomplish. To be able to take a good impression and to put a high finish on rubber plates is a very small part of the requirements of a prosthetic artist. One of the most important features, namely, "taking the bite," is almost always considered of comparatively little consequence. We once knew of a celebrated dentist spending three or four long sittings with a patient in taking the bite and modeling the feature in the wax bite so as to get a faultless bite and an artistic result. The next most important feature is not the selection of properly formed and colored teeth, though this is of very great importance, but to articulate the teeth so that the patient will get the full value of every tooth in mastication. In our early history this feature was deeply studied and strongly advocated, but it fell almost into disuse until revived and scientifically studied by the late Dr. Bonwill. Just at this time there seems to be a growing interest in this direction which promises hopeful results for our profession from the prosthetic standpoint, or department. It is claimed by the orthodontists that no one devoid of artistic sensibilities, or who does not possess them in a very high degree, ought to undertake to regulate teeth. We feel that the same might very appropriately be said of the practitioners of prosthetic dentistry. If they do not possess this instinct, they should certainly endeavor to cultivate it or drop out this class of work entirely.

Dr. T. G. Rix: Forty years ago we had artistic men in the profession as a rule, instead of as an exception, as now. This was due to the fact that it required brains and skillful handicraft to perform the prosthetic requirements of those days. The introduction of vulcanite as a base plate superseded the metal plates then universally used, and there has been a gradual decline or demoralization from which we must recover, if there be any practicable improvement in methods.

Dr. Cook: I find that almost invariably patients want to dictate in the selection of the teeth or in the manner in which they are to be adjusted. They want white and small teeth set in rows; the more even and uniform the more like they are to approve of the teeth even though they may not fit, and we are compelled from the strong competition to fall into commercialism. It is right that we should have an ideal and that we should strive to attain it in every case; but there are so many diverting experiences, that it is practically impossible to give the time and thought, and to get the patient's co-operation for attaining our ideals.

Dr. S. W. Honey: If we have not known our patient before the teeth were lost it may be difficult to make a restoration which will suit the patient or his friends. I recently had a case which to my mind was a very satisfactory restoration, but it did not please my patient's friends and had to be made over in accordance with their suggestions before it was accepted.

Dr. Warboys, in closing: There is need of some fixed and definite data by which we may get such fundamental facts as color and size of teeth. These may be made in accordance with artist's standards, but are not so satisfactory as to have some measurements or a plaster model of the teeth of the patient before extracting had been done. Such a record will be found to be of very great help in selecting and articulating the teeth.

